# Combinatorial screening of halide perovskite thin films and solar cells by mask-defined IR laser molecular beam epitaxy

**DOI:** 10.1080/14686996.2017.1314172

**Published:** 2017-04-28

**Authors:** Kazuhiro Kawashima, Yuji Okamoto, Orazmuhammet Annayev, Nobuo Toyokura, Ryota Takahashi, Mikk Lippmaa, Kenji Itaka, Yoshikazu Suzuki, Nobuyuki Matsuki, Hideomi Koinuma

**Affiliations:** ^a^ Fundamental Research Department, Comet Co. Ltd, Tsukuba, Japan; ^b^ Graduate School of Pure and Applied Sciences, University of Tsukuba, Tsukuba, Japan; ^c^ The Technology Centre of Academy of Science and Technology, Ashgabat, Turkmenistan; ^d^ Institute for Solid State Physics, University of Tokyo, Chiba, Japan; ^e^ Japan Science and Technology Agency, JST PRESTO, Saitama, Japan; ^f^ North Japan Research Institute for Sustainable Energy, Hirosaki University, Aomori City, Japan; ^g^ Department of Electrical, Electronics and Information Engineering, Faculty of Engineering, Kanagawa University, Yokohama-shi, Japan; ^h^ National Institute for Materials Science, NIMS, Tsukuba, Japan

**Keywords:** Combinatorial deposition, inorganic–organic hybrid material, halide perovskite solar cell, IR laser MBE, 40 Optical, magnetic and electronic device materials, 209 Solar cell / Photovoltaics, 306 Thin film / Coatings

## Abstract

As an extension of combinatorial molecular layer epitaxy via ablation of perovskite oxides by a pulsed excimer laser, we have developed a laser molecular beam epitaxy (MBE) system for parallel integration of nano-scaled thin films of organic–inorganic hybrid materials. A pulsed infrared (IR) semiconductor laser was adopted for thermal evaporation of organic halide (A-site: CH_3_NH_3_I) and inorganic halide (B-site: PbI_2_) powder targets to deposit repeated A/B bilayer films where the thickness of each layer was controlled on molecular layer scale by programming the evaporation IR laser pulse number, length, or power. The layer thickness was monitored with an *in situ* quartz crystal microbalance and calibrated against *ex situ* stylus profilometer measurements. A computer-controlled movable mask system enabled the deposition of combinatorial thin film libraries, where each library contains a vertically homogeneous film with spatially programmable A- and B-layer thicknesses. On the composition gradient film, a hole transport Spiro-OMeTAD layer was spin-coated and dried followed by the vacuum evaporation of Ag electrodes to form the solar cell. The preliminary cell performance was evaluated by measuring *I*-*V* characteristics at seven different positions on the 12.5 mm × 12.5 mm combinatorial library sample with seven 2 mm × 4 mm slits under a solar simulator irradiation. The combinatorial solar cell library clearly demonstrated that the energy conversion efficiency sharply changes from nearly zero to 10.2% as a function of the illumination area in the library. The exploration of deposition parameters for obtaining optimum performance could thus be greatly accelerated. Since the thickness ratio of PbI_2_ and CH_3_NH_3_I can be freely chosen along the shadow mask movement, these experiments show the potential of this system for high-throughput screening of optimum chemical composition in the binary film library and application to halide perovskite solar cell.

## Graphical abstract

**Figure:**
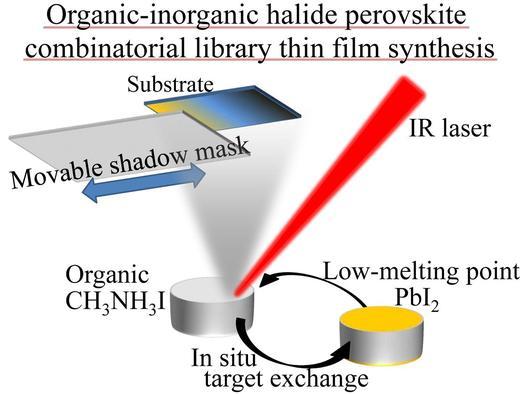

## 1. Introduction

Halide perovskites, ABX_3_ (X=I, Br, Cl) have attracted much interest, ever since the demonstration of high photovoltaic energy conversion efficiency of methylammonium lead iodide: CH_3_NH_3_PbI_3_ (MAPbI_3_) cells [1]. This halide or organic–inorganic hybrid perovskite solar cell has fascinating physical properties [2–7] and there was a rapid increase of conversion efficiency from 3.8% in 2009 to 22.1% in March 2017 [8]. A major limitation of perovskite solar cells is the low stability of the crystal lattice and rapidly degrading cell performance [9–11]. Attempts to improve the properties of the halide materials have been undertaken; however, conventional one-by-one experiments [2,12–17] are very time consuming for exhaustive screening of the possible parameter space.

In this work, we approach this problem by high-throughput screening of cell compositions and synthesis parameters for optimizing the device performance. We have developed a combinatorial IR laser MBE system and report preliminary results on the application of the high-throughput techniques to the fabrication of halide perovskite thin films and solar cell libraries. This thin film growth system design was derived from earlier pulsed UV and continuous wave (CW) IR laser MBE systems that have been developed for depositing oxide ceramic [18–21] and organic [22,23] thin films, respectively. We demonstrated that the digitally controlled deposition of molecular layers by coupling IR laser evaporation with a computer controlled mask action could successfully locate the perovskite phase in the composition gradient film library. Even by defining just seven 2 mm × 4 mm solar cell devices within a lateral composition spread library, the optimum test device exhibited a solar energy conversion efficiency of 10.2%.

## 2. Experimental details

The first step of this study was designing and setting up a new combinatorial film deposition chamber that uses a CW IR laser for digitally controlled evaporation of halide perovskite precursor compounds. The chamber is also equipped with a motorized shadow mask manipulator that is used to selectively open only a part of the substrate to the evaporation flux. Synchronizing the evaporation laser pulses with the mask movement is used to control the thickness of each individual deposited layer in either a stepped or continuous gradient pattern over the substrate surface, forming a combinatorial library. A halide perovskite MAPbI_3_ combinatorial library was fabricated by a multilayer deposition [24] of the precursor materials: CH_3_NH_3_I (MAI) and PbI_2_, with a controlled layer thickness by mask action in the IR laser MBE system. The perovskite phase was confirmed by X-ray diffraction (XRD) and ultraviolet-visible (UV-vis) absorption spectroscopy. A combinatorial solar cell device was fabricated with a spin-coated hole transport layer, followed by vacuum evaporation of Ag electrode, on the perovskite library film and *I*-*V* characteristics were measured to evaluate the cell performance.

### 2.1. Design of the combinatorial IR laser MBE system

**Figure 1.**
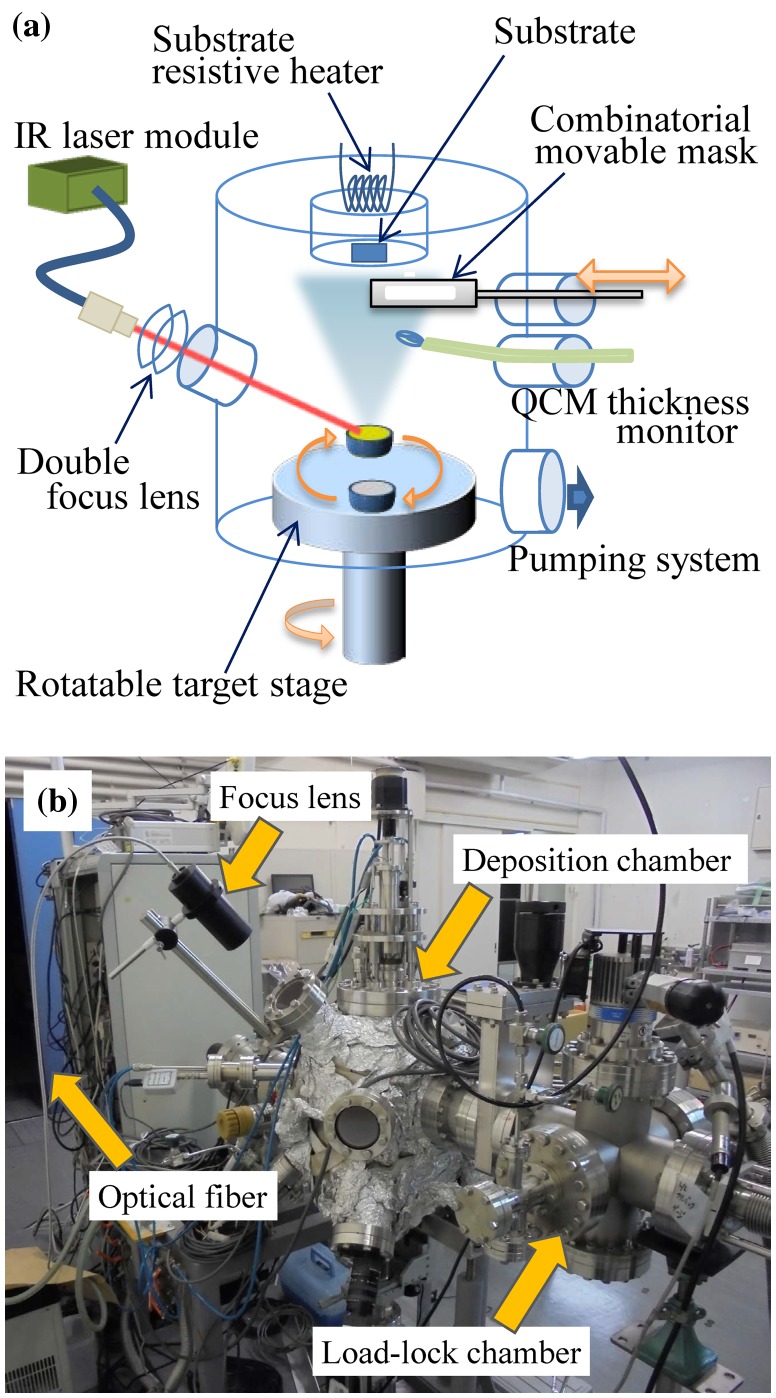
(a) Schematic diagram of the IR laser MBE system with a single IR laser source. A combination of the single IR laser and the rotatable target stage enables an evaporation right below the substrate position, thus the deposited composition is well defined, unlike a co-deposition method. (b) A photograph of the combinatorial IR laser MBE system.

The combinatorial film deposition system was specially designed for the nano-scaled fabrication of organic–organic and organic–inorganic hybrid layers to construct a binary phase diagram and mixed alloys. There are two ways for the binary phase diagramming, i.e. the fabrication of composition-gradient films of two source materials. One is the so-called composition spread method, originating from spontaneous sputtering of multi targets [25,26], and the other is the alternating stacking of a pair of nano-layers of two target materials by using a single evaporation or ablation source at a time and a movable shadow mask to obtain the desired layer thickness or composition gradient in the lateral direction [26]. The latter method was chosen for this work due to several advantages over a co-deposition scheme. Instead of UV excimer laser used for pulsed laser ablation of solid oxides, a semiconductor IR laser was employed for the evaporation of volatile organic and hybrid materials. A schematic and a photograph of the combinatorial IR laser MBE system are shown in Figure 1. The system is equipped with a rotatable target stage and a movable mask for combinatorial synthesis. A DirectBond 800 (DirectPhotonics, Germany) semiconductor laser operating at 808 nm and a maximum power of 30 W was used for thermal evaporation of soft film precursors such as organic MAI and inorganic PbI_2_, similar to an earlier work on organic molecules [22]. The IR laser beam was transferred from the laser module to a view port of the vacuum chamber by an optical fiber. Fiber collimator and focus lenses were placed in front of the view port to focus the laser on the target. A target reagent was put in a small Pyrex glass beaker with an inner diameter of 15 mm. In the current system, up to four beakers can be mounted on a rotatable target stage that enables target exchange during the deposition. Owing to the target exchange system, the following advantages are obtained. Unlike a traditional MBE system with individual heaters and shutters for each evaporation source, only a single heating laser with a fixed focus point was needed to sequentially evaporate any of the up to four source materials. During evaporation, the active source is directly facing the substrate, which means that unwanted deposition rate gradients that occur with offset sources in typical MBE systems can be avoided. This deposition technique is thus fundamentally different from co-evaporation techniques where evaporation fluxes from two or more sources overlap at the substrate position at an angle, producing a natural composition spread film [27,28] where additional *ex situ* analysis is necessary to determine the precise composition. The second advantage of the target exchange system is that the hetero and multilayer structures can be easily grown thanks to accurate control over the thickness of deposited layer, as described below. These advantages directly contribute to the increased throughput of material development and device performance optimization.

A substrate was placed upside down above the target stage as shown in Figure 1(a). The substrate can be heated up to 500°C by a resistive heater mounted on the backside of the substrate holder. A quartz crystal microbalance (QCM) was installed to evaluate the real-time thickness of the deposited layer that was calibrated in advance by an *ex situ* measurement with a stylus profilometer.

The deposition system was equipped with a combinatorial movable mask that was mounted in front of the substrate at a distance of about 1 mm. The mask enables us to grow combinatorial library samples where the thickness of a particular layer or the chemical composition varies continuously over the surface of a sample [20,21,29,30].

### 2.2. Combinatorial library synthesis and characterization

**Figure 2.**
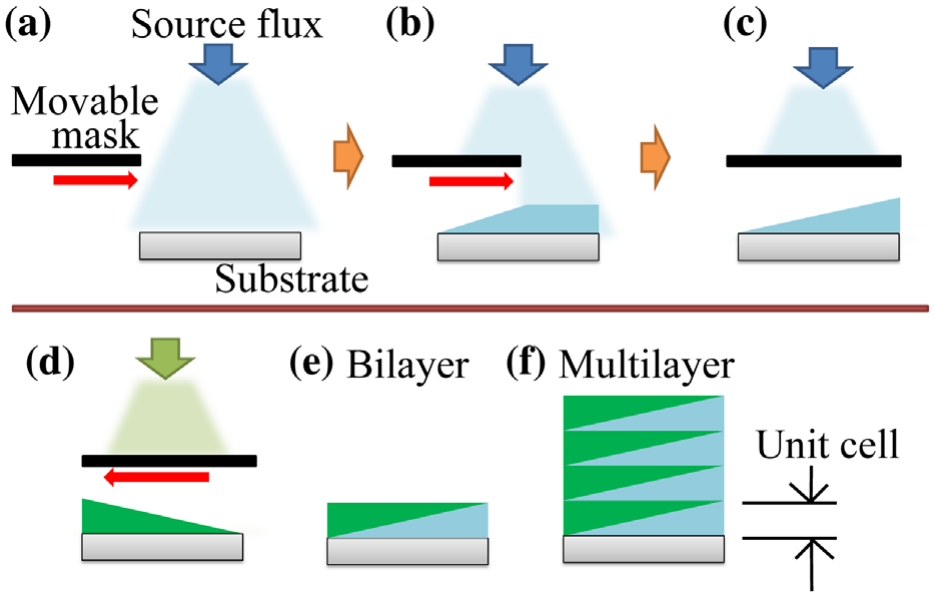
Illustration of the combinatorial gradient deposition method. (a–c) By moving a shadow mask in front of the sample during deposition, a layer thickness gradient is obtained. (d) The gradient direction can be selected by using the opposite edge of the mask. Combining two layers with opposite gradient direction produces a flat layer with a spatially varying composition, known as a composition spread (e). A thick film can be grown by repeating the deposition of bilayers (f).

The combinatorial film growth method is illustrated in Figure 2. By moving the mask during the deposition (Figure 2(a) and 2(b)), it is possible to grow a film that has a controlled thickness gradient (Figure 2(c)). The gradient direction can be selected by using one or the other edge of the mask (Figure 2(d)). A flat film with spatially varying composition can be grown by combining two layers with opposite gradient directions (Figure 2(e)). A film with programmed composition variation can be obtained by repeating the deposition of bilayers (Figure 2(f)). By characterizing the film properties within a single combinatorial sample, multiple data points can be systematically acquired. Note that one combinatorial library sample gives a continuous phase diagram according to the controlled parameter. The method of combinatorial library formation as shown in Figure 2 can be extended to the screening of a ternary phase library by a combinatorial mask with a triangle slit [21].

In this work, two target reagents, PbI_2_ and MAI (Wako Pure Chemical Industries, Japan) in the Pyrex glass beakers were placed on the rotatable target stage. Each reagent was mixed with Si polycrystalline powder to support absorbance of the IR laser light. The base pressure of the deposition system was 1.1 × 10^−5^ Pa, and the substrate temperature was 26°C during film growth. The IR evaporation laser was modulated at 3 Hz with a pulse length of 200 μs. The laser power for the evaporation was about 2.2 W for PbI_2_ and 3.2 W for MAI on average.

Focusing the IR laser on the MAI target, MAI is evaporated and MAI film forms on the substrate with the thickness of the film being proportional to the period that a particular part of the substrate is exposed by the movable mask. Rotating the stage to focus the laser beam on PbI_2_ target, thickness gradient PbI_2_ film can be deposited in the same way. Switching the two targets in synchronization with the IR laser and masking give a MAI and PbI_2_ bilayered film. A multilayered structure consisting of a stack of thin PbI_2_ and MAI layers was grown, which transformed into perovskite MAPbI_3_ according to a chemical formula CH_3_NH_3_I + PbI_2_ → CH_3_NH_3_PbI_3_ during the deposition. Synthesis of the MAPbI_3_ perovskite phase by depositing PbI_2_/MAI multilayers has been reported before [24]. In that report, vacuum evaporation with K-cells was used to grow PbI_2_(50 nm)/MAI (50 nm) multilayers that were allowed to react. In our work, we deposited PbI_2_/MAI multilayers with a much finer structure, where each component layer thickness was in the order of a unit cell. An important advantage of the finer structure is that the stoichiometry in the growth direction is guaranteed to be homogeneous due to a MAI diffusion into PbI_2_ layer [31]. Note that the combinatorial library fabricated in this way possesses a homogeneous stoichiometry in the vertical direction and the controlled composition gradient in the horizontal direction by the combinatorial mask.

A PbI_2_/MAI multilayer film was grown on a 12.5 × 12.5 × 1.1 mm^3^ indium tin oxide (ITO) coated glass substrate (GEOMATEC, Japan). The growth started with the deposition of a PbI_2_ layer with a thickness of 1.4 nm, which corresponds to two unit cells along the *c*-axis direction, and then a MAI layer on top of the PbI_2_ layer. The thickness of the MAI layer was controlled from 0 nm at one edge of the sample to 3.4 nm at the opposite edge by using the movable shadow mask. The growth of PbI_2_ (1.4 nm)/MAI(0–3.4 nm) bilayer was repeated 300 times, yielding a combinatorial library with a built-in MAI thickness gradient in a single thin film sample and sufficient cumulative thickness for XRD analysis and UV-visible absorption spectroscopy.

XRD measurements were performed with a Bruker D8 DISCOVER diffractometer. In order to obtain multiple data points from one combinatorial sample, the X-rays from the generator were confined to a circular spot with a diameter of 1 mm. In this manner, 11 positions were measured in a combinatorial library sample along the gradient direction. UV-vis spectroscopy was carried out with a V-570 spectrophotometer (JASCO, Japan). A pinhole slit with a diameter of 1.5 mm was used. Position dependence of the surface morphology in the combinatorial library was measured with a SU8230 scanning electron microscope (SEM) (Hitachi, Japan).

### 2.3. Fabrication of combinatorial solar cell device and characterization

**Figure 3.**
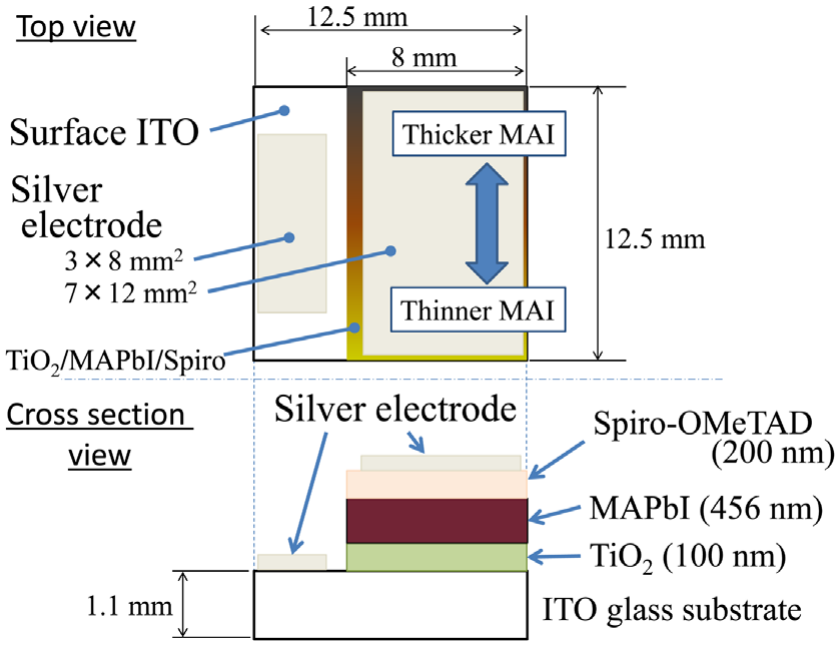
Structure of a combinatorial solar cell library used for mapping composition-dependent device characteristics.

A combinatorial PbI_2_/MAI solar cell device was fabricated. The structure of the device is shown in Figure 3. A compact TiO_2_ film was used as the electron transport layer. The MAPbI_3_ layer was the light absorber, and a 2,2′,7,7′-tetrakis (N,N-di-p-methoxyphenylamine)-9,9′-spirobifluorene (Spiro-OMeTAD) layer functioned as the hole transport layer. Silver metal electrodes were used for contact. The compact TiO_2_ and Spiro-OMeTAD layers were grown by spin coating. Details of the electron and hole transport layer growth can be found in [32]. The combinatorial technique was applied to the solar cell fabrication as well. A [PbI_2_(1.4 nm)/MAI(0–3.4 nm)]×300 multilayer was deposited on the TiO_2_-coated ITO glass substrate. The silver electrodes were evaporated on top by conventional resistive thermal evaporation. The conversion efficiency was measured by recoding *I*-*V* characteristic under AM1.5 simulated solar light (100 mW cm^–2^ – XES-40S1, SAN-EI Electric, Japan). To perform *I*-*V* measurements on the combinatorial library device, seven shadow masks were used to limit the 2 × 4 mm^2^ area exposed to light. *I*-*V* characteristic could thus be measured at seven different positions on the library. The voltage range of the *I*-*V* scans was –0.1 V to 1.1 V in both forward and reverse directions. The voltage step and delay time were 20 mV and 50 ms. We did not set a pre-illumination time, and the illumination was started 2 s before the measurement. Cross-sectional SEM image was used to verify that the solar cell device structure matched the design parameters.

## 3. Results and discussion

**Figure 4.**
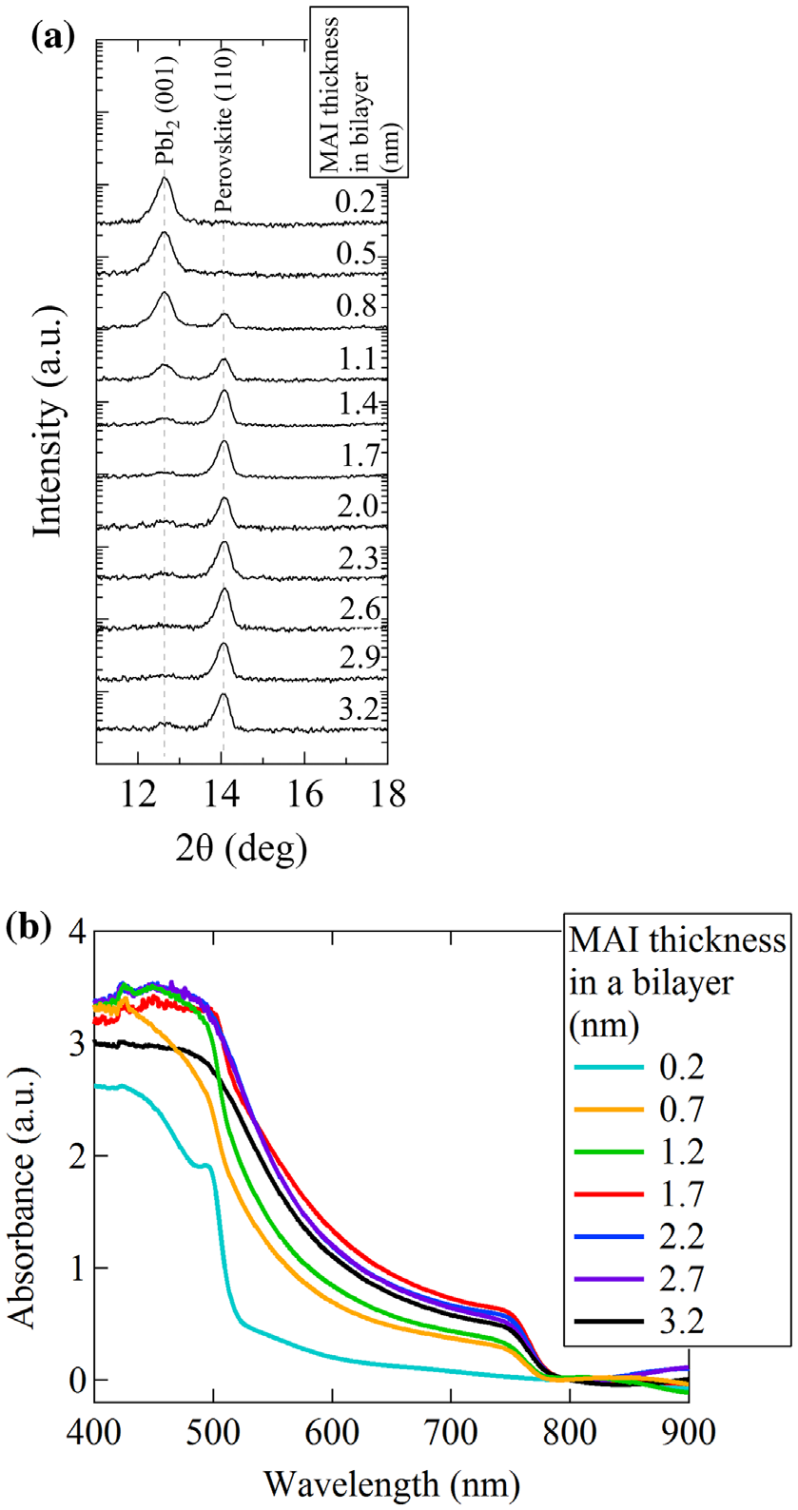
(a) XRD *θ*-2*θ* scans of a composition spread library. The (001) PbI_2_ diffraction peak and the (110) perovskite MAPbI_3_ peak are at 12.63° and 14.05°, respectively. Each curve corresponds to a measurement at a different position in the combinatorial composition-spread library. The MAI thickness in a bilayer increases from top to down, and the PbI_2_ layer thickness was kept at 1.4 nm. The numeric layer thickness values correspond to an average in the area illuminated by X-rays. (b) UV-visible absorption spectra of the combinatorial library.

Combinatorial XRD *θ*-2*θ* scans of the MAPbI_3_ composition gradient library on an ITO substrate are shown in Figure 4(a), focusing on the low angle region from 2*θ*=11–18°. The diffraction peaks at 12.63° and 14.05° correspond to the (001) PbI_2_ peak and the (110) MAPbI_3_ perovskite peak, respectively. It is clear that the (110) MAPbI_3_ peak does not exist at the edge of the library with the smallest MAI layer thickness. The (110) MAPbI_3_ diffraction peak intensity is enhanced as the MAI ratio increases in the film and, at the same time, the PbI_2_ diffraction peak intensity is suppressed. When the MAI layer thickness exceeds 1.4 nm, the PbI_2_ peak becomes invisible, indicating a full reaction of PbI_2_ to the perovskite phase. The (110) MAPbI_3_ peak intensity was nearly constant for MAI layer thicknesses of more than 1.4 nm because the volume of the MAPbI_3_ crystal does not change even when excess MAI is present.

UV-visible absorption spectra of the combinatorial library are shown in Figure 4(b). At the position where the MAI layer thickness is 0.2 nm, an absorption feature corresponding to the bandgap of PbI_2_ appears at ~510 nm. As the MAI ratio increases in the film, the absorption associated with PbI_2_ vanishes. Instead, absorption due to the MAPbI_3_ phase at ~790 nm gradually increases. When the MAI layer thickness reaches 1.7 nm, the MAPbI_3_ absorption enhancement reaches a maximum. Although the spectrum is hardly influenced by an increase of the MAI thickness beyond 1.7 nm, a small suppression of absorbance is observed at the position in the library where the MAI thickness was 3.2 nm.

The XRD and UV-vis results clearly demonstrate that the PbI_2_ precursor phase reacts gradually and forms the MAPbI_3_ perovskite phase in the combinatorial library. We succeeded in observing a systematic change of the structural and optical properties as a function of the thickness of the MAI layer.

**Figure 5.**
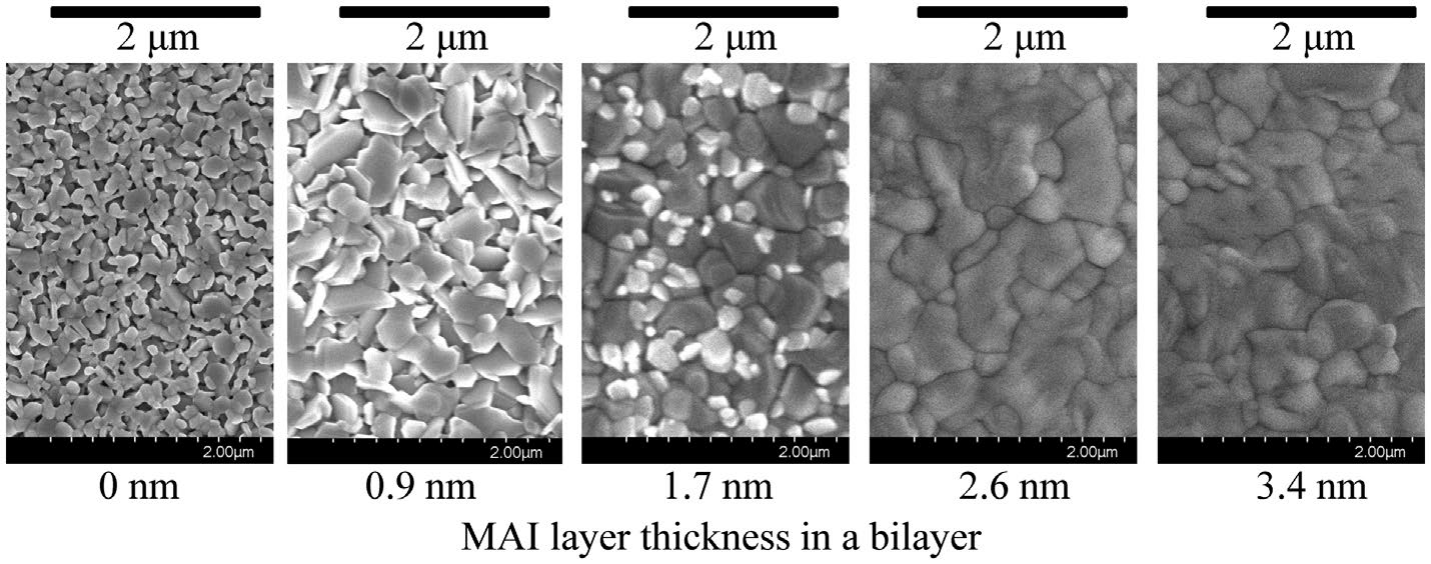
Position dependence of the film surface morphology in the combinatorial library, measured by SEM. The MAI layer thickness increases from left to right.

Figure 5 shows the surface SEM images taken at different positions in the combinatorial library. It is clear that the grain size becomes larger as the MAI layer thickness increases. This grain swelling is associated with the crystal growth process of MAPbI_3_ [31].

**Figure 6.**
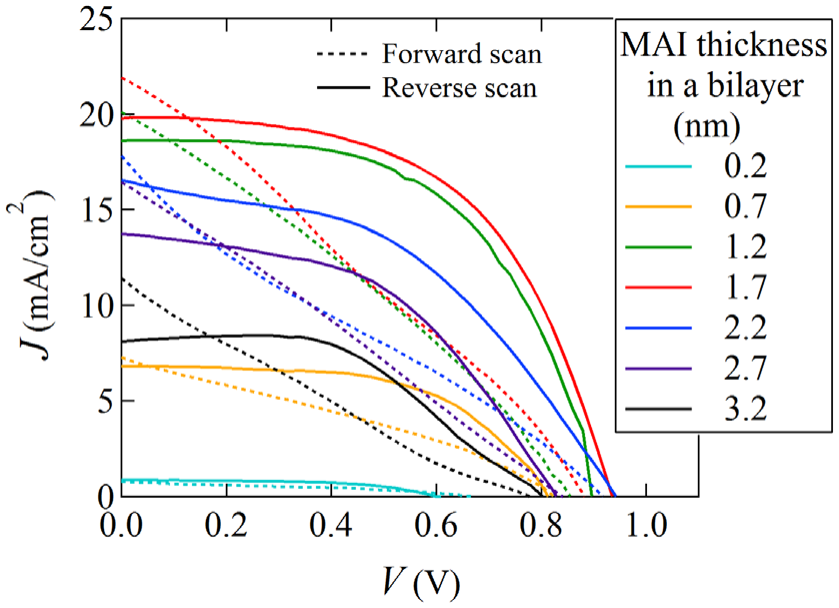
The *I*-*V* characteristics of a combinatorial solar cell device library. Film composition ratios are shown for each measured library position in the legend. Data from forward and reverse bias scans are plotted with dashed and solid lines, respectively.

The *I*-*V* mapping results of the combinatorial device are shown in Figure 6. The seven curves correspond to different positions in the library measured with the shadow masks. It is clear that the PbI_2_ rich region and the MAI rich region show suppressed *J*_SC_ and *V*_OC_. Note that our solar cell device shows a large hysteresis, which may be attributed to a ferroelectric effect, trapping of carriers at the interface, or ionic movement [33–35].

**Figure 7.**
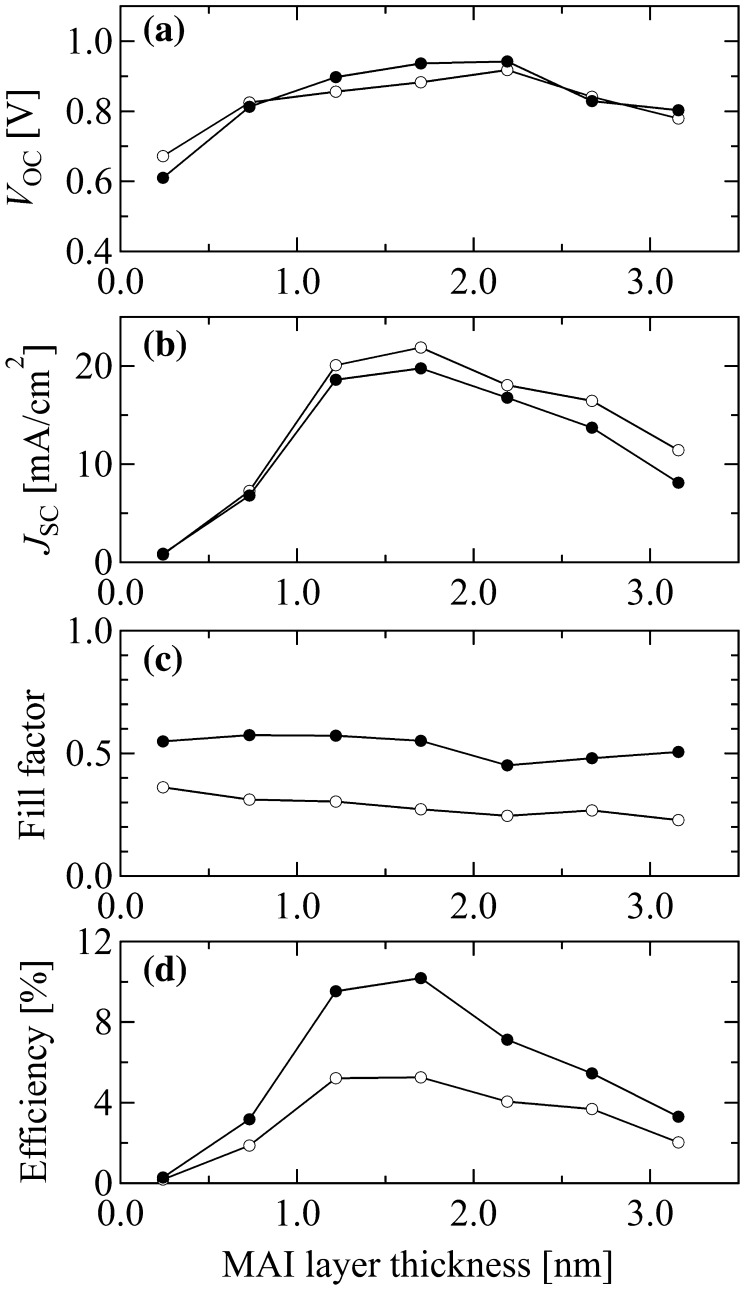
*V*_OC_ (a), *J*_SC_ (b), fill factor *FF* (c) and conversion efficiency *η* (d) as a function of the MAI layer thickness in a single combinatorial solar cell library. The open and solid data points correspond to the values from forward and reverse bias scans, respectively.

To conduct a more detailed analysis, four parameters: short-circuit current density *J*_SC_, open-circuit voltage *V*_OC_, fill factor (*FF*) and conversion efficiency *η*, were extracted from the *I*-*V* characteristics. Figure 7 summarizes the dependences of *J*_SC_, *V*_OC_, *FF* and *η* on the MAI thickness in forward and reverse bias scans. The plots show that *V*_OC_ and *J*_SC_ are approximately comparable between the forward and reverse scans. However, there is a gap in the *FF* data caused by the hysteresis of the *I*-*V* characteristics and a reduction of *η* in the forward bias scan. In the PbI_2_ rich region, *J*_SC_ is strongly suppressed, dropping to about 1 mA cm^–2^. As the MAI thickness increases, *J*_SC_ increases and reaches a maximum of 21.9 mA cm^–2^ and 19.2 mA cm^–2^ in the forward and reverse scans, respectively, at a position that corresponds to a PbI_2_ (1.4 nm)/MAI(1.7 nm) bilayer. Due to excess MAI beyond this point, *J*_SC_ is suppressed again. The reduction of *J*_SC_ indicates that excess PbI_2_ or MAI give rise to recombination centers that suppress the current density. *V*_OC_ shows a similar tendency, although the variation is less pronounced than for *J*_SC_. Especially at around the region where the MAI thickness is ~1.7 nm, *V*_OC_ variation is negligible. The fill factor was not sensitive to the film composition. As a result, the power conversion efficiency, *η*, reached a maximum of 5.3% and 10.2% in the forward and reverse scans, respectively, when the bilayer composition was PbI_2_ (1.4 nm)/MAI(1.7 nm), indicating that this thickness ratio gives a film stoichiometry with the lowest recombination losses.

It has been demonstrated that the magnitude of the hysteresis is influenced by defect concentration in the perovskite film in conjunction with the grain size of the perovskite crystal [36]. Grain growth offsets the defect concentration in the film, resulting in stable power output of the solar cell device. From this viewpoint, the hysteresis observed in the library device can have two origins. One is the excess PbI_2_ or MAI. They exist in the perovskite film as impurities, contributing to an increase of the interstitial defect concentration and enhancing hysteresis [37]. Note that this explanation does not demonstrate the hysteresis observed in a nominally stoichiometric composition of [PbI_2_ (1.4 nm)/MAI (1.7 nm)]×300. The other possible cause for hysteresis is related to the small size of the perovskite crystalline domains. Domains with a size less than 170 nm are known to cause a severe hysteresis [38]. Average perovskite domain sizes in the library were calculated from the SEM images in Figure 5 as 194, 239, 252, 439, and 451 nm from left to right. Note that the average grain size of [PbI_2_ (1.4 nm)/MAI (1.7 nm)]×300 (the image at the center in Figure 5) is 252 nm, which is larger than 170 nm. However, as can be seen in the SEM image, the grains at this position are composed of those with various sizes, including small grains with a diameter of down to ~90 nm. Therefore, those small grains increase the defect concentration in the film, resulting in hysteresis in the *I*-*V* measurements.

**Figure 8.**
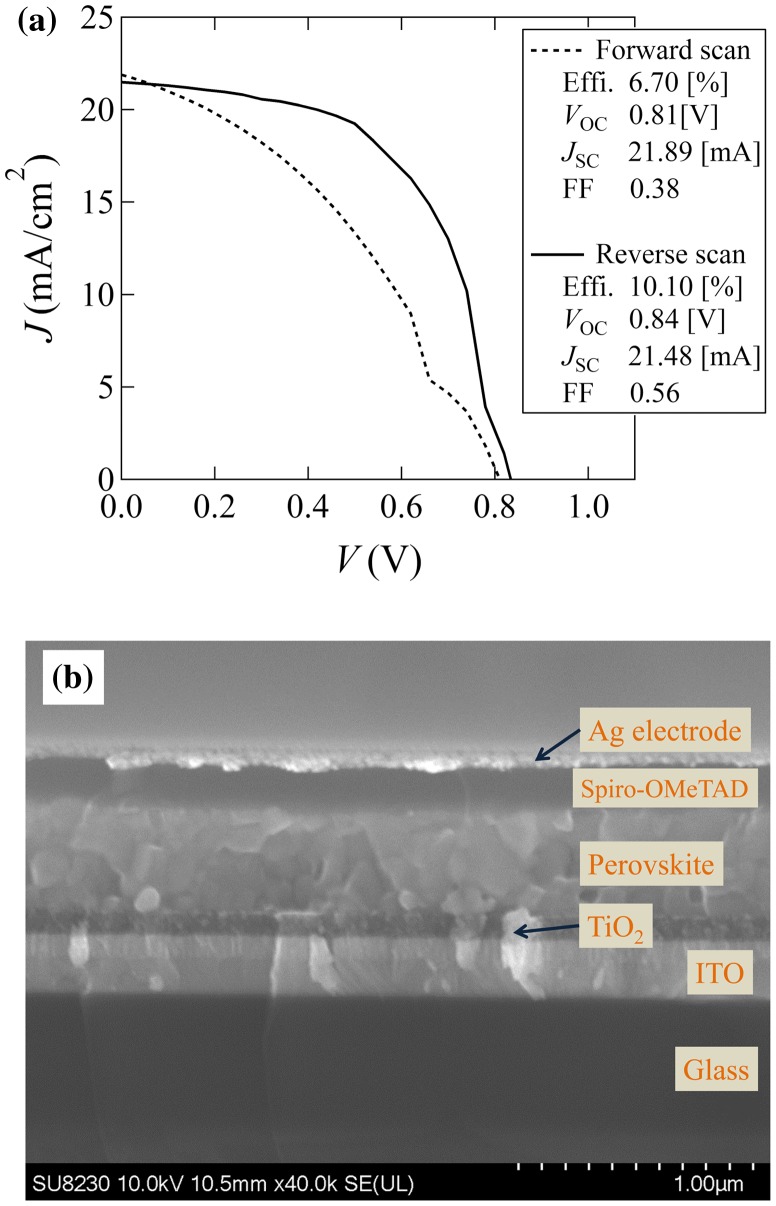
(a) *I*-*V* characteristic of the solar cell device with a homogeneous [PbI_2_ (1.4 nm)/MAI(1.7 nm)]×300 multilayer film as the absorbing layer. (b) Cross-sectional SEM image of the solar cell device.

A solar cell device with a homogeneous [PbI_2_ (1.4 nm)/MAI(1.7 nm)]×300 absorbing layer was fabricated in order to evaluate the possibility of practical application of the combinatorial technique. The *I*-*V* characteristic of the device is shown in Figure 8(a). The conversion efficiency of this device was comparable to that expected from the result of the combinatorial library screening, demonstrating the validity of the combinatorial screening technique for the hybrid halide perovskite compound and device development. A cross-sectional SEM image of this solar cell device is shown in Figure 8(b). The boundary of each component layer is sharp and well defined.

**Figure 9.**
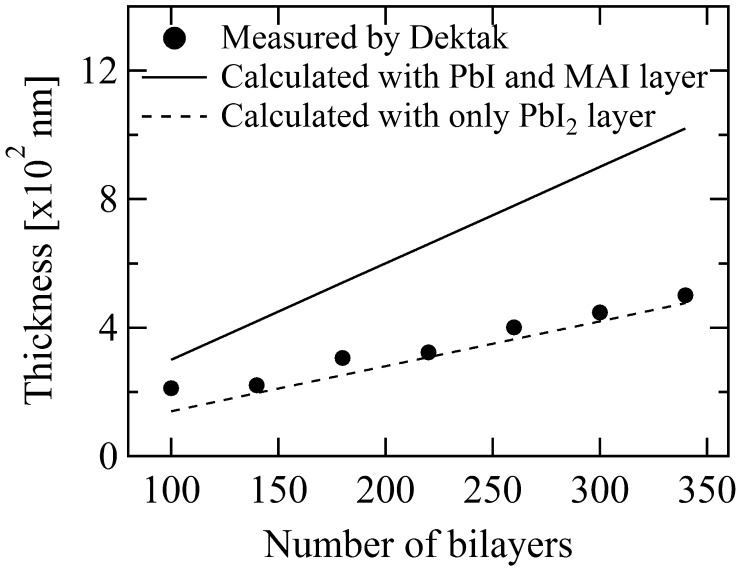
Relation between the measured total film thickness and the calculated estimates for a combinatorial thickness gradient perovskite MAPbI_3_ film. The solid line is a thickness estimate based on all deposited layers (PbI_2_ and MAI). The dashed line presents an estimate based on the PbI_2_ layer thickness only.

So far, the thickness of the perovskite MAPbI_3_ film was controlled by the number of bilayer repetitions. Another MAPbI_3_ library was grown for total film thickness calibration. In this library, the composition of the bilayers was fixed at PbI_2_ (1.4 nm)/MAI(1.7 nm), but the repetition number was varied from 100 to 340 times across the sample area. The film thickness was measured with a Dektak stylus profilometer (Vecco, USA) at seven positions, as shown in Figure 9. The solid line in the plot corresponds to the nominal total thickness, calculated by multiplying a single bilayer thickness of 1.4 nm + 1.7 nm by the repetition number. The dashed line corresponds to a thickness calculated from the PbI_2_ layer alone, multiplying the PbI_2_ thickness of 1.4 nm by the repetition number. It is interesting to note that the measured thickness is close to the PbI_2_ thickness rather than total PbI_2_+MAI estimate. This apparent discrepancy can be explained by the fact that CH_3_NH_3_I and PbI_2_ react chemically, and one unit cell of PbI_2_ and one unit cell of MAI form one unit cell of the MAPbI_3_ perovskite. From the measured relation between the MAPbI_3_ thickness and the bilayer repetition number, the thickness of the perovskite layer in the solar cell device at the position where the PbI_2_/MAI ratio is 1.4 nm/1.7 nm, is determined to be 445.5 nm. This thickness value is consistent with previous reports where the thicknesses of the absorber layer are generally in the range of 300–600 nm.

## 4. Conclusions

We have developed an IR laser MBE system for organic–inorganic hybrid materials. Pulsed IR laser evaporation of volatile precursors has the advantage of accurate layer thickness and deposition rate control. We fabricated PbI_2_/MAI multilayers, where the thickness of each layer was controlled with an accuracy of a unit cell. The system is equipped with a movable mask that can be used for combinatorial deposition of composition or thickness gradient libraries. XRD measurements, UV-visible spectroscopy, and surface SEM images showed a systematic conversion from the PbI_2_ precursor phase to the MAPbI_3_ perovskite phase as the thickness of the MAI layer was increased.

Solar cell device fabrication was integrated with the combinatorial perovskite libraries, allowing efficient mapping of device characteristics as a function of film composition. A strong suppression of the conversion efficiency was observed for both PbI_2_ rich and MAI rich compositions. The highest efficiency of 10.2% was obtained in a combinatorial solar cell library at the position where the ratio of the PbI_2_/MAI component layer thicknesses was 1.4 nm/1.7 nm. The measured thickness of the reacted perovskite layer in the device at this position was 445.5 nm. As a result, we have confirmed that the structural and electronic properties of PbI_2_/MAI multilayers strongly depend on the PbI_2_/MAI stoichiometry. Systematic changes of device performance characteristics were observed by combinatorial library mapping, indicating that the combinatorial technique is applicable to the synthesis of halide perovskite thin films.

The extension of the combinatorial technique to the organic–inorganic hybrid material was successfully demonstrated. In the combinatorial technique, physical parameters such as chemical composition, layer thickness, and substrate temperature are intentionally controlled to obtain a combinatorial library, which accelerates not only a material exploration but also optimization of deposition conditions and a device structure. Although there are reports of using laser MBE for the growth of hybrid perovskite compounds [31,39,40], the successful demonstration of combinatorial screening in this work shows that the basic laser MBE method can be further improved to accelerate the development of organic–inorganic hybrid materials. In addition to materials synthesis, the combinatorial technique can also increase the efficiency of optimizing perovskite solar cell device structures that have several important components such as the electron and hole transport layers, a Si/perovskite tandem design [41], etc., where the structure and the choice of recombination layer is still a subject of intense research. Moreover, further efficient screening technology can be established by a collaborative combination of experimental and first principle screening. These projects are in progress, and the development of the combinatorial IR laser MBE system for the hybrid perovskite material reported in this paper is the first concrete step towards that goal.

## Disclosure statement

No potential conflict of interest was reported by the authors.

## Funding

The work was supported in part by JSPS Kakenhi [grant number 26105002] and JST PRESTO.
